# Sustainable capital funding for modern and innovative radiotherapy services

**DOI:** 10.1093/bjr/tqaf255

**Published:** 2025-10-21

**Authors:** Imogen Powell Brown, Daniel Hutton, Nicola Thorp, James Thomson, Ran MacKay, Liesl Hacker, Lisa Ashmore, John Hayes, John Archer, Carl Rowbottom

**Affiliations:** NW Radiotherapy Specialised Services Clinical Network, Manchester M20 4BX, United Kingdom; NW Radiotherapy Specialised Services Clinical Network, Manchester M20 4BX, United Kingdom; Lancaster University, Lancaster LA1 4YW, United Kingdom; The Christie, Manchester M20 4BX, United Kingdom; The Clatterbridge Cancer Centre, Liverpool L7 8YA, United Kingdom; The Christie, Manchester M20 4BX, United Kingdom; The Christie, Manchester M20 4BX, United Kingdom; Lancaster University, Lancaster LA1 4YW, United Kingdom; Cheshire and Merseyside Cancer Alliance, Liverpool LA1 4YW, United Kingdom; The Christie, Manchester M20 4BX, United Kingdom; The Clatterbridge Cancer Centre, Liverpool L7 8YA, United Kingdom

**Keywords:** capital expenditure, capital replacement, linear accelerators, linacs, radiotherapy funding, advanced radiotherapy, radiotherapy demand

## Abstract

Radiotherapy providers are dependent on capital investment in equipment, which makes up 62% of the cost of delivering radiotherapy. The commissioning of radiotherapy services and duty to replace equipment is currently held by NHS England, but this responsibility and funding will be delegated to all Integrated Care Boards (ICBs). With constraints in national health capital spend over the past decade leaving radiotherapy infrastructure depleted, ICBs are set to inherit an expensive task of updating and replacing radiotherapy equipment. The upcoming National Cancer Plan presents the opportunity for a long-term solution to the renewal and investment in radiotherapy equipment, through rolling ringfenced funding from Government.

This paper is part of a series of three papers, on (1) radiotherapy tariff, (2) radiotherapy capital spending and (3) holistic aspects of radiotherapy funding, which together consider what a sustainable, innovative and person centred radiotherapy funding model looks like as specialised services are delegated to Integrated Care Boards.

## State of radiotherapy equipment

Radiotherapy a highly effective curative and palliative treatment for cancer through the targeting of tumours. Delivery depends on advanced imaging scans used to accurately identify cancer tumours and Linear Accelerators (LINACs), which provide most external radiotherapy treatments to patients. These technologies are capital dependent with equipment accounting for 62% of total radiotherapy costs.^1^ High initial costs of LINACs are realised over a 10-year period and can achieve a positive return on investment when considering wider economic costs of cancer^2^ and, overall, radiotherapy remains a cost-effective cancer treatment, accounting for only 7% of the total cancer spend.^1^ Ensuring reliable funding for these resources is needed to maintain the quality and accessibility of radiotherapy services for patients across England.

Insufficient UK Government investment in NHS capital costs over the past decade is well documented.^3–7^ During the 2010s, a schism between UK capital spending levels and other countries opened up. Had the UK matched capital investment of its peers this would have amounted to an additional £37 billion.^2^ This spending could have eliminated the entire capital backlog maintenance in England which currently stands at around £11.6bn.^2^

Low investment has left the radiotherapy equipment supply depleted. The United Kingdom consistently ranks below the Organisation for Economic Co-operation and Development (OECD) average for the number of CT, MRI and PET scans.^8^ England has 4.8 LINACs per million inhabitants,^9^ placing it behind the European average of 5.9 and towards to lower end of the estimated required number of 4.0 to 8.1.^10^ Across England, radiotherapy departments are using out of date LINACS and there is no national coordinated replacement scheme in place.^1^ LINACs have a recommended life span of 10 years; older machines lack imaging capabilities for modern treatments and are slower and have increased down time negatively impacting on productivity and utilisation.^11^ The Royal College of Radiologists suggests that 36% of cancer centres in England reported that their equipment breaks down most months, which will have repercussions on waiting times for patients and service productivity.^12^

Effective LINAC utilisation requires comprehensive funding from providers for equipment maintenance costs and software costs, alongside capital investment to replace equipment. However, the tariff system which remunerates providers for day-to-day running has also failed to keep up to date with the rising costs associated with modern radiotherapy delivery.^1^ Importantly, sufficient capacity within the radiotherapy workforce is needed to improve access radiotherapy,^13^ however, in England, the NHS radiographer workforce has a vacancy rate of around 8.43% indicating deficiencies in workforce planning, particularly within recruitment and retention.^14^

## Delegation to integrated care boards

Radiotherapy providers experience the front-line cost of outdated radiotherapy equipment and along with the 11 radiotherapy Specialised Services Clinical Networks (SSCN) *formally Operational Delivery Networks (ODN), have a responsibility to develop a replacement programme for LINACs. However, they have limited control in funding capital replacement schemes.

Providers have previously been required to submit their multiyear replacement plans to NHS England. However, as Integrated Care Boards (ICBs) inherit greater responsibility of the commissioning of radiotherapy services through the delegation of specialised services, all providers will instead submit plans to ICBs. ICBs will fund these plans through their system operational capital allocations, whilst the specialised services budget devolved from NHSE will be absorbed into this ICB budget without the requirement that expenditure for radiotherapy equipment is ringfenced.

ICBs are currently reporting financial strain with around three quarters of the 42 integrated care systems unable to set balanced budgets for 2024-25,^15^ raising the risk that the radiotherapy budget will be absorbed into already stretched ICBs funding priorities. In August 2023, 70% of ICB’s had no named person responsible for ensuring access to sufficient treatment capacity for radiotherapy.^16^

The approach runs counter to calls for a centralised radiotherapy replacement programme.^11^^,^^17^ Long term funding could support ICBs to implement a strategic approach to replacing machines. While the £70 m of funding announced for radiotherapy machines at the latest budget was welcome,^18^ as were similar injections of funding in 2016 and 2020,^19^^,^^20^ they did not provide permanent financial security to providers. Unplanned injections of funding from Government make systems disincentivised to save or prepare for replacement of LINACs as they come to the end of their life span. In Scotland there has been a government funded equipment replacement programme for radiotherapy since 1999 which supports the ongoing replacement of treatment-related equipment, covering LINACs and wider technologies needed such as Treatment Planning Systems and Record and Verify Systems.^21^ The 2022 Radiotherapy Action Plan set aside a £45million rolling ringfenced fund for this scheme which allows radiotherapy centres to plan and tender for replacement when LINACs reach the end of their 10- year life cycle.^22^

## Supporting a modern and innovative radiotherapy service

ICBs will need to respond to emerging developments in radiotherapy equipment to support an advanced and innovative radiotherapy service. ‘Standard’, image guided LINACs, which are in main use have cone beam CT capability whereas more 'Innovative’, next generation LINACs, might incorporate advanced imaging, automation and the ability for real time adaptation of radiotherapy planning and delivery. For example, the MR-LINAC, which are not formally commissioned by the NHS, combine an MRI scanner and LINAC to locate and tailor the shape of X-ray beams in real time to treat moving tumours.^23^ This technology has the potential support the accurate delivery of radiotherapy treatment and limit side effects for patients. ICBs can facilitate the adoption of more innovative equipment across the country, however, the upfront capital costs compared with ‘standard’ LINACs create challenge for ensuring the equitable spread of these technologies across the country.

Meanwhile, the price of LINAC machines has risen significantly in recent decades,^24^ with the ability of providers to set aside their own budgets for the replacement of machines often depleted by broader constraints in radiotherapy funding.

## Conclusion

In 2040, 30% more cancer cases are expected to be diagnosed each year in the UK.^25^ National demand modelling, with regional breakdowns, such as provided by the Malthus model can be used to determine the full radiotherapy activity this could lead to.^26^ Appropriate funding from the UK Government is needed for patients to receive equitable and timely access to high quality radiotherapy treatment in coming years.

The upcoming National Cancer Plan for England gives an opportunity to provide long term sustainability within radiotherapy funding. As upfront costs for ICBs increase, assurance is needed from the Government that funding will is available for LINAC replacement and related infrastructure, through a national rolling ringfenced fund. ICBs would be responsible for submitting tender requests to national teams ahead of their LINACs coming to the end of their lifespan. The setting of the central fund should be reflective of inflationary trends and be able to respond to requests for modern machines which have higher costs than traditional LINACS. Central coordination and oversight of replacing LINACs should be set up to support equitable distribution of more innovative machines.

Wider reform to the radiotherapy tariff and workforce planning is needed so that investment in equipment results in a more productive and cost effective service for patients. As the delegation of specialised services goes ahead across England, funding arrangements and expectations of individual providers, the Radiotherapy Specialised Service Clinical Delivery Networks and ICBs need to be clearly articulated from the national level.

Further research could estimate the current population demand for radiotherapy, reflecting advances in techniques such as hypofractionation, rather than relying on current activity levels. This could determine the size and scope of radiotherapy equipment required, rather than just replacing existing capacity, and define the workforce that would be required to support this. Previous estimates show that although an estimated 40%-50% of cancer patients will require radiotherapy as part of their treatment, access rates to radiotherapy in England are around 37%.^27^ Further complexity is added to this as radiotherapy capacity does not currently reflect regional variations in cancer incidence.^27^
